# Results of a portfolio approach to intramural research funding at an academic medical center

**DOI:** 10.1371/journal.pone.0241425

**Published:** 2020-11-06

**Authors:** Anu Swaminathan, Frank S. David, Lauren N. Geary, Jacqueline M. Slavik

**Affiliations:** 1 Brigham Research Institute, Brigham and Women's Hospital, Boston, Massachusetts, United States of America; 2 Pharmagellan LLC, Milton, Massachusetts, United States of America; Charles P. Darby Children's Research Institute, UNITED STATES

## Abstract

In response to stagnant Federal grant funding levels and to catalyze early stage or high-risk research not currently supported by the NIH, many academic medical centers (AMCs) provide supplemental intramural funding to faculty investigators. However, it can be challenging to decide how to deploy these funds for maximum impact. We conducted a retrospective, descriptive analysis to explore trends in applications and awards associated with an institution-wide intramural funding center at a major U.S. AMC. From 2010 to 2017, the Brigham Research Institute at Brigham and Women’s Hospital awarded a total of 354 grants totaling over $9 million to affiliated researchers through six distinct and complementary grant programs. The number of applicants remained essentially stable, despite expansion of the funding program portfolio. Distribution of applicants and awardees by academic rank and gender generally reflected that of medical school faculty at large. This descriptive analysis demonstrates interest in a diverse range of intramural funding programs among AMC faculty, and a lack of overt rank or gender bias in the programs’ awardees. However, it highlights the institution’s need to better understand the amount of residual unmet demand for intramural funding; the degree to which underrepresented constituencies can and should be actively supported; and the “return on investment” of these grants.

## Introduction

Research is a core component of the mission of U.S. academic medical centers (AMCs), but its viability in the 21^st^ century has been threatened by a progressive decline in extramural funding. In inflation-adjusted dollars, appropriations from the National Institutes of Health have stagnated since peaking in 2003, and over the same period, the success rate for R01-equivalent awards has declined by about a third [1]. These trends have made it difficult for physician-scientists [2] and other AMC researchers to initiate and advance their careers.

Partly in response to this burdensome extramural funding environment, many AMCs provide intramural research funding to schools, departments, and individual investigators. A recent study reported that for every dollar of external research sponsorship, AMCs spent about 50 cents of internal funds on research. Although almost 70 percent of this AMC investment went toward facilities and administrative subsidies and salary support, about 10 percent was provided directly to investigators in the form of bridge funding or university (intramural) research grants [3].

Beyond deciding *whether* to provide internal research funds to investigators and *how much* to spend, however, AMCs must decide *how* to deploy these supplemental resources. AMCs often design and implement new programs based on a qualitative assessment of the institution’s overall goals, the likely impact of the various options under consideration, and the amount of resources available. But when budgets come up for renewal, program administrators typically face pressure to quantitatively gauge these programs’ effectiveness in order to justify to senior executives which ones should be maintained, expanded, or terminated. The rigor of this assessment is often limited by the quality of the underlying data, small sample sizes, and inherent difficulties in defining relevant outcomes that can be measured on relatively short time scales.

In this study, we report the results of a descriptive, retrospective analysis of internal research funds disbursed by a dedicated center within a large U.S. AMC. In 2005, Brigham and Women’s Hospital (BWH), a top-20 recipient of NIH funding among academic medical centers, launched the Brigham Research Institute (BRI). The BRI’s mandate includes increasing the visibility of research at BWH, bolstering communication within the research community, catalyzing internal and external collaborations, and aiding in fundraising. Starting in 2007, the BRI began directing a substantial portion of its resources toward providing supplemental research funding to internal investigators, using funds from its largely philanthropically supported operating budget. This decision was made partly in response to administrators’ view that the challenging extramural funding environment was restricting the research careers of BWH researchers at all levels, from trainees to senior investigators. Supplemental research funding was also seen as an opportunity to further support the BRI’s main goals according to its mandate (see above). Under the guidance of the institute’s oversight and executive committees, the BRI opted to fund a broad variety of programs, targeting a diverse range of research endeavors and constituencies, instead of a narrowly defined set of grant types, sizes, and/or applicant ranks.

We undertook the study reported here to assess two aspects of the BRI’s portfolio of intramural grants. First, what are the demographics of grant applicants and recipients, and to what extent do they highlight areas where further attention and activity may be warranted? And second, how have application numbers changed over time, and what does this tell us about the demand for intramural funding opportunities? With some caveats (discussed below), both aims were achieved.

## Materials and methods

### Data collection

We reviewed BRI’s internal records on the number of applications and awards, as well as dollars disbursed, for each funding mechanism from 2010 to 2017. For all mechanisms except microgrants (for which complete data were not available), we also identified each applicant’s and awardee’s academic rank and gender from application materials, supplemented as needed with data from BWH’s institutional directory and direct outreach to applicants. We were able to assign gender for over 99% (480/483) of applicants and all awardees, and academic rank for 100% of both applicants and awardees. We assigned each applicant a unique numerical identifier to enable us to publicly release anonymous data.

Data on the gender and academic rank distribution of full-time faculty at U.S. medical schools from 2010 to 2017 (Faculty Roster, December 31 snapshots as of January 31, 2020) were provided to the investigators by the American Association of Medical Colleges, Washington, DC, USA.

### Data analysis

Spearman correlations were calculated using Microsoft Excel. Chi-squared analyses were performed in Microsoft Excel, using templates developed by Missouri State University (https://www.missouristate.edu/rstats/Tables-and-Calculators.htm). All reported p values are two-sided. Because all analyses were exploratory and hypothesis generating, adjustments for multiple hypothesis testing were not performed.

### Ethics approval

This study was based on administrative data that did not include patient contact or medical record review, and therefore did not require institutional review board approval or informed consent.

## Results

From 2010 to 2017, the BRI awarded a total of 354 grants totaling over $9 million through six intramural funding mechanisms, supported by institutional resources and generous contributions from private donors. These six funding programs reflect an intentional blend of award size and focus (Table 1). The Fund to Sustain Research Excellence (FSRE) provides fairly conventional small bridge grants, intended to cover gaps in research dollars in the window between missed R01 paylines and approval in subsequent NIH grant cycles. Initiated to tackle head-on the challenge of declining R01 funding rates, the FSRE has maintained steady popularity since its inception. Center-specific research grants are offerings (variable in award amount, frequency, and specific goals) by the BRI’s individual centers, each of which is focused on a specific research domain or disease area. These grants aim to seed collaborations within their respective communities or provide pilot funding for special priorities identified within them. The “Shark Tank” program is similar in process to other institutions’ efforts to support “bench to bedside” investigations [4], and further bolsters the BRI’s mandate to increase donor engagement by involving them in determining the final winners. Like the Shark Tank, the BRIght Futures prizes target innovative science with practical ramifications that may fall outside of what is traditionally funded by the NIH. BRIght Futures also serves the BRI’s mission of increasing research visibility, because the final recipient each year is decided by an online vote, open to the lay public worldwide [5]. Finally, the BRI has two programs that target extreme ends of the funding spectrum: microgrants support low-cost endeavors, mainly related to professional development and the seeding of new research directions; whereas the Directors’ Transformative Awards are large grants to ground-breaking collaborative endeavors. Although each program has specific application requirements, most are open to faculty and staff at all levels, with more limited eligibility for postdoctoral researchers.

**Table 1:** Applicants, awards, and funds awarded through BRI funding programs, 2010–2017.

Funding program name	Description		2010	2011	2012	2013	2014	2015	2016	2017	Total
**Total**	All funding sources	Applicants	15	86	171	225	126	197	128	158	1,106
		Awards	12	18	69	66	45	45	46	53	354
		Funds awarded	$520K	$900K	$981K	$1.22M	$1.14M	$1.43M	$1.25M	$1.62M	$9.06M
**Fund to Sustain Research Excellence**	Bridge funding for grants that miss NIH R01 payline ($50K)	Applicants	12	26	19	11	18	15	16	25	142
		Awards	10	12	10	7	16	11	10	17	93
		Funds awarded	$500K	$600K	$500K	$350K	$800K	$550K	$500K	$700K	$4.50M
**Center-Specific Research Grants**	Research grants aligned with specific scientific or clinical areas (up to $50K)	Applicants	3	2	17	72	N/A	30	4	8	136
		Awards	2	2	2	14	N/A	2	2	5	29
		Funds awarded^a^	$20K	$100K	$100K	$620K	N/A	$100K	$20K	$90K	$1.05M
**Shark Tank**	Funding for projects that could impact biomedical research, healthcare delivery, the generation of new companies/products/services, cost savings, care quality, or provider burnout ($50K)	Applicants	N/A	58	44	69	69	60	36	40	376
		Awards	N/A	4	4	5	4	3	2	4	26
		Funds awarded	N/A	$200K	$200K	$100K	$200K	$150K	$100K	$200K	$1.15M
**BRIght Futures**	Awards for projects that “[catalyze] the kind of innovative translational research that is only possible at an academic medical center” ($100K)	Applicants	N/A	N/A	9	15	10	20	15	14	83
		Awards	N/A	N/A	1	1	1	1	1	1	6
		Funds awarded	N/A	N/A	$100K	$100K	$100K	$100K	$100K	$100K	$600K
**Microgrants**	Grants to defray expenses for career development, training, and other research-related endeavors (up to $2,500)	Applicants	N/A	N/A	82	58	29	34	36	45	284
		Awards	N/A	N/A	52	39	24	25	30	24	194
		Funds awarded^a^	N/A	N/A	$81K	$46K	$38K	$32K	$33K	$29K	$259K
**Directors’ Transformative Awards**	Funding for “cross-departmental, interdisciplinary activities that support collaborative projects” (up to $500K)	Applicants	N/A	N/A	N/A	N/A	N/A	38	21	26	85
		Awards	N/A	N/A	N/A	N/A	N/A	3	1	2	6
		Funds awarded	N/A	N/A	N/A	N/A	N/A	$500K	$500K	$500K	$1.50M

Dollar figures may not sum precisely due to rounding. Abbreviations: N/A, not applicable because funding mechanism was inactive during that year. K, thousand. M, million.

^a^ Rounded to nearest $1,000.

### Gender and academic rank of BRI grant applicants and awardees

We analyzed the characteristics of applicants and awardees across all of the funding mechanisms except for microgrants, for whom we lacked the associated full demographic information. Over the eight-year analysis period, these programs received 822 applications from 483 unique individuals. Of the applicants, 36% (173/483) applied for more than one grant in the same year and/or different years (range among the 173 “multi-appliers,” 2–11; median, 3; interquartile range, 2–3). “Multi-appliers” were more likely to receive funding than individuals who applied just once (44% vs. 18%, p<0.00001 by Chi-squared test). Compared with one-time applicants, “multi-appliers” were equally likely to be female (66% vs. 64%, p = 0.57 by Chi-squared test), but more likely to be at the instructor or assistant professor level at the time of their first award (92% vs. 68%, p<0.00001 by Chi-squared test).

Over the same period, we awarded 160 grants via these programs to 133 unique individuals. Among the awardees, 83% (110/133) received just one BRI grant, 15% (20/133) received two awards, and the remainder (3/133) received 3–4 grants apiece. Due to the small number of recipients who received more than one award, we elected not to perform statistical tests to compare these individuals to the single-grant awardees.

We performed three analyses to explore the gender and academic rank distributions of applicants and awardees, using a standard two-sided p value cutoff of 0.05 to define statistical significance. First, we compared the characteristics of individuals who submitted applications to those who received awards over the entire eight-year period. There was not a statistically significant difference in the fraction identifying as female between these two groups (285/822 applicants (35%) vs. 53/160 awardees (33%); p = 0.71 by Chi-squared test). Similarly, when we analyzed by faculty rank, excluding research fellows and other non-faculty titles, there was no difference in the percent of faculty at the more junior levels of instructor and assistant professor between the two groups (561/808 applicants (69%) vs. 104/160 awardees (65%); p = 0.27 by Chi-squared test).

Second, we compared the genders and academic ranks of applicants versus awardees in each individual year (Tables 2 and 3). In 2016, we observed a statistically significant difference between applicants and awardees in the percent of individuals identifying as female (23/92 (25%) vs. 8/16 (50%); p = 0.041 by Chi-squared test). No other differences between applicants and awardees with regard to academic rank or gender in individual years were statistically significant.

**Table 2:** Comparison of fraction of females among BRI grant applicants, BRI grant awardees, and U.S. medical school faculty by year, 2010–2017.

Year	Female applicants	Female awardees	Female U.S. medical school faculty	p values (χ^2^)		
				Applicants vs. awardees	Applicants vs. U.S. faculty	Awardees vs. U.S. faculty
**2010**	8/15 (53%)	5/12 (42%)	52,299/144,619 (36%)	0.55	0.17	0.69
**2011**	40/86 (47%)	8/18 (44%)	55,502/150,978 (37%)	0.87	0.061	0.50
**2012**	24/89 (27%)	2/17 (12%)	57,830/154,691 (37%)	0.18	0.042	0.029
**2013**	66/167 (40%)	9/27 (33%)	60,977/159,846 (38%)	0.54	0.71	0.61
**2014**	33/97 (34%)	5/21 (24%)	64,310/165,364 (39%)	0.36	0.33	0.16
**2015**	52/163 (32%)	4/20 (20%)	67,505/170,511 (40%)	0.28	0.045	0.073
**2016**	23/92 (25%)	8/16 (50%)	70,317/174,538 (40%)	0.041	0.0028	0.43
**2017**	39/113 (35%)	12/29 (41%)	73,046/177,629 (41%)	0.49	0.15	0.98

U.S. medical school statistics are for faculty at all ranks (including “other”).

**Table 3:** Comparison of proportion of BRI grant applicants, BRI grant awardees, and U.S. medical school faculty at junior academic rank by year, 2010–2017.

Year	Junior applicants	Junior awardees	Junior U.S. medical school faculty	p values (χ^2^)		
				Applicants vs. awardees	Applicants vs. U.S. faculty	Awardees vs. U.S. faculty
**2010**	9/15 (60%)	8/12 (67%)	52,299/144,619 (36%)	0.72	0.71	0.43
**2011**	64/86 (74%)	12/18 (67%)	55,502/150,978 (37%)	0.50	0.00054	0.36
**2012**	66/89 (74%)	12/17 (71%)	57,830/154,691 (37%)	0.76	0.00058	0.23
**2013**	123/160 (77%)	19/27 (70%)	60,977/159,846 (38%)	0.46	1.58 x 10^−7^	0.14
**2014**	70/96 (73%)	16/21 (76%)	64,310/165,364 (39%)	0.76	0.0014	0.073
**2015**	103/160 (64%)	12/20 (60%)	67,505/170,511 (40%)	0.70	0.059	0.79
**2016**	57/91 (63%)	11/16 (69%)	70,317/174,538 (40%)	0.64	0.28	0.35
**2017**	69/111 (62%)	14/29 (48%)	73,046/177,629 (41%)	0.18	0.27	0.35

“Junior rank” defined as Instructor or Assistant Professor (versus Associate Professor or Full Professor); see text for details.

Third, we compared applicants and awardees by year to full-time faculty members of U.S. medical schools, as reported by the American Association of Medical Colleges (see Methods). Compared with the U.S. faculty, the BRI applicant pool had a smaller percentage of females in three of the eight years, and a larger fraction of individuals at junior ranks in four of the years. Among BRI awardees, females were under-represented compared with the national faculty in 2012. All other gender and academic rank comparisons with U.S. medical school faculty were not statistically significant.

### Trends in grant applications over time

We examined trends in application numbers by year to the BRI’s funding programs using Spearman’s rank order calculations (Table 4). We excluded center-specific research grants, which had highly variable program execution and design across years that led to vast differences in application numbers. The remaining grant programs accounted for 88% (970/1,106) of the applications received from 2010 to 2017. Our analysis failed to detect a statistically significant trend in applications over time to all programs taken together (r_s_ = 0.40, p = 0.40) or to any of the programs individually.

**Table 4:** Spearman rank-order correlations for BRI funding programs over time.

Funding mechanism	Spearman coefficient (r_s_)	t statistic	p value
All funding mechanisms*	**0.40**	**0.91**	**0.40**
BRI Directors’ Transformative Awards	-0.50	-1.1	0.33
BRIght Futures Prize	0.41	0.92	0.39
Fund to Sustain Research Excellence	0.12	0.29	0.78
Microgrants	-0.43	1.2	0.28
Shark Tank	-0.40	-0.90	0.40

Source data on applicant numbers per year for each funding mechanism taken from Table 1. *Excluding center-specific research grants; see text for details.

## Discussion

In this study, we analyzed the number and type of applicants and awardees across several distinct research funding mechanisms at the BRI, a provider of substantial intramural support to investigators at a major AMC. Our goals were to assess (1) the gender and academic rank distribution of grant applicants and awardees in comparison to each other and benchmark data on U.S. medical school faculty, and (2) the growth in applications over time as an indicator of demand for these sorts of funding programs.

With regard to demographics, we did not detect a significant bias by gender or rank when comparing awardees to applicants. Our applicant pool did skew slightly less female and more junior than U.S. medical center faculty overall in several years, but most of these differences were not present when we compared awardees to national faculty overall. With regard to applications over time, we did not observe a statistically significant change in the number of applications over time. Of note, all of these results should be considered exploratory and hypothesis generating only, as they were neither pre-specified nor adjusted for multiple hypothesis testing.

Although the demographic data presented here suggest that the BRI’s grant awards are neither intentionally nor unintentionally biased with regard to female gender, the slight under-representation of women in the applicant pool in several years compared with the national sample is notable and worthy of further investigation. Structural bias is known to impede the career development of female medical faculty, and although mentorship programs and other activities have been somewhat successful in combating it, women remain under-represented in academic medicine, particularly at senior ranks [6, 7]. Although some evidence suggests that grant funding may not be a critical component of the broader gender imbalance [8], data are scant in this area. Furthermore, aside from a recent initiative sponsored by Australia’s National Health and Medical Research Council, there have been few efforts to explicitly promote gender equity in academic medicine with funding programs that specifically target female faculty [9, 10]. Intramural funding groups like the BRI could explore similar programs to boost faculty diversity.

Our analysis of the number of applications over time failed to demonstrate an increase in demand for the BRI’s funding programs. This was somewhat surprising, given that over the same period the BRI increased the diversity of funding mechanisms and engaged in a significant degree of outreach and awareness-building, and the number of faculty grew over this time as well. These results several possibilities that merit investigation: demand may be adequately met by the current intramural grant offerings, and/or outreach efforts to date may have been ineffective at boosting awareness of these funding opportunities, and/or investigators may not find the programs appealing based on the size of the awards, the odds of receiving them, and the effort and time required to apply. In the meantime, our analysis does not currently support the need to increase the breadth, diversity, or number of awards offered by the BRI in order to satisfy demand.

This study illuminates two further areas related to intramural research funding that warrant additional study. First, it is not clear whether grant programs like the BRI’s can and should do more to support broader diversity goals in academic medicine with regard to race and ethnicity. The leadership of BWH is philosophically committed to promoting racial and ethnic diversity within the research community, which has long been known to be lacking [11–13]. In 1989, NIH began offering research supplements to minority investigators as part of a broader suite of initiatives to combat systemic under-representation of particular groups [14]. However, we have been unable to identify any systematic assessment of the impact on diversity of NIH’s minority faculty funding program, and a recent longitudinal study suggested that across most specialties, representation of blacks and Hispanics has actually decreased over the past two decades [15]. To date the BRI has not explicitly focused its funding programs on furthering diversity, and because the BRI has not systematically collected race and ethnicity data from applicants and awardees, we were unable to assess these factors in the current analysis. However, we plan to obtain and analyze these data going forward as we assess how we may be able to deploy intramural funding in a way that promotes faculty diversity.

Second, this work implicitly highlights challenges of evaluating the impact of intramural funding programs in academic medicine. Although the BRI is not required to achieve a specific performance threshold to continue its programs, the question of return on investment (ROI) is top of mind for our leadership team, executives, and potential donors. We have explored applying ROI analysis methods similar to those used by other institutions to assess their intramural career development programs [16–18], but we have been hindered by the low quantity and quality of data we have been able to obtain from awardees, as well as significant outliers that make statistical analyses difficult. We are currently re-evaluating how we track outcomes after grant awards in order to enable more rigorous studies in the future of the impact of these awards on relevant metrics of career success and development.
